# Pancoast tumor presenting with multiple joint pains: a case report

**DOI:** 10.1186/s13256-022-03328-4

**Published:** 2022-03-15

**Authors:** Safia Mohamud, Mosunmola Oyawusi, Roger L. Weir, Etuajie O. Halbert, Richard M. Millis, Teddy Gebremedhin, Ozra Dehkordi

**Affiliations:** 1 Department of Neurology, George Washington University Hospital, Washington, DC 20037 USA; 2 Department of Neurology, Howard University Hospital, Washington, DC USA; 3 Department of Psychiatry, Howard University Hospital, Washington, DC USA; 4 Department of Pathophysiology, College of Medicine, American University of Antigua, Coolidge, Antigua Antigua and Barbuda

**Keywords:** Pancoast tumor, Polyarthritis, Superior sulcus tumors

## Abstract

**Background:**

Pancoast tumors represent a unique subset of lung cancers wherein a primary neoplasm arises in the lung’s apex and invades the surrounding soft tissues. One of the main challenges in the diagnosis and treatment of these apical lung cancers is that they are usually not visualized on initial chest x-ray and, by the time the patient presents with symptoms, the tumor has almost always invaded nearby structures.

**Case presentation:**

Herein we report a case of a 58-year-old nonsmoking African American male who presented to the neurology clinic with a history of multiple chronic joint pains. The patient complained of shoulder pain that traveled into his right arm and right finger and had worsened over the past 9 months. The patient also reported decreased right proximal strength and swelling of his right hand. Magnetic resonance imaging of the shoulder and cervical region showed mild cervical spondylosis and a questionable right apical mass. A subsequent high-resolution computed tomography scan of the chest revealed a large right apical lung mass, with chest wall invasion and erosion of the adjacent ribs. Biopsy of the mass confirmed poorly differentiated non-small cell lung cancer. Radiation therapy was initiated, and the patient’s pain improved significantly. Given the size of the tumor, chemotherapy was recommended by the oncology team. The patient decided against chemotherapy.

**Conclusion:**

This case highlights the importance of early diagnosis by expanding the differential diagnosis in patients presenting with weakness, sensory loss, and shoulder pain beyond radiculopathy or joint-related diseases. A comprehensive history and careful examination may lead to an earlier diagnosis, more appropriate treatment, and better outcome in cases of Pancoast tumor presenting with neuropathic or musculoskeletal pain.

## Background

Pancoast tumors, also known as superior sulcus tumors, account for less than 3–5% of all lung carcinomas [1–4]. Pancoast tumors are located in the apex of the lung, are poorly visualized on chest x-rays, and by the time they are discovered, have already invaded different compartments of the thoracic inlet including brachial plexus, vertebral bodies, and subclavian vessels [4]. The clinical syndrome associated with Pancoast tumors, named “Pancoast–Tobias syndrome,” involves severe shoulder and arm pain along the distribution of the eighth cervical and first and second thoracic nerve trunks, associated with atrophy of the hand and arm muscles, as well as Horner’s syndrome [1–6]. Delay in detection on initial chest x-ray and difficulties in the initial differential diagnosis pose special challenges to neurologists, oncologists, and radiologists.

## Case presentation

A 58-year African American male with multiple joint pains had hip replacement 5 years previously and was being followed by orthopedics for chronic hip pain. The patient was referred to our neurology clinic for worsening of right upper extremity (RUE) weakness and numbness over the past 9 months. He reported back pain that traveled into his right arm and included a burning sensation from the right shoulder down to the right hand, with constant nighttime awakenings. The pain was described as electric in quality, shooting down the right arm to the right fingertips. He also reported decreased right proximal strength and swelling of the right hand.

**Fig. 1:**
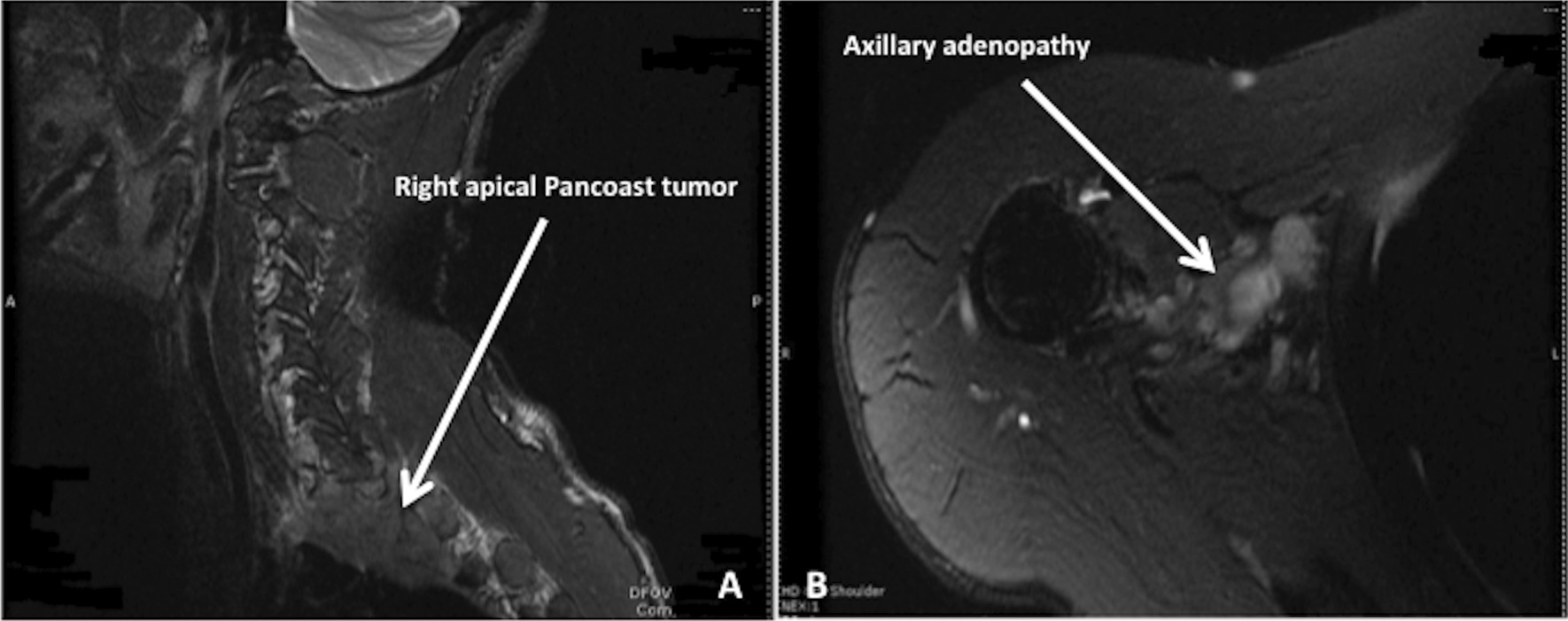
**A** Cervical spine MRI showing right apical mass (arrowhead) and mild cervical spondylosis. **B** Right shoulder MRI showing glenohumeral joint effusion (arrowhead). The rotator cuff and labrum are unremarkable

**Fig. 2:**
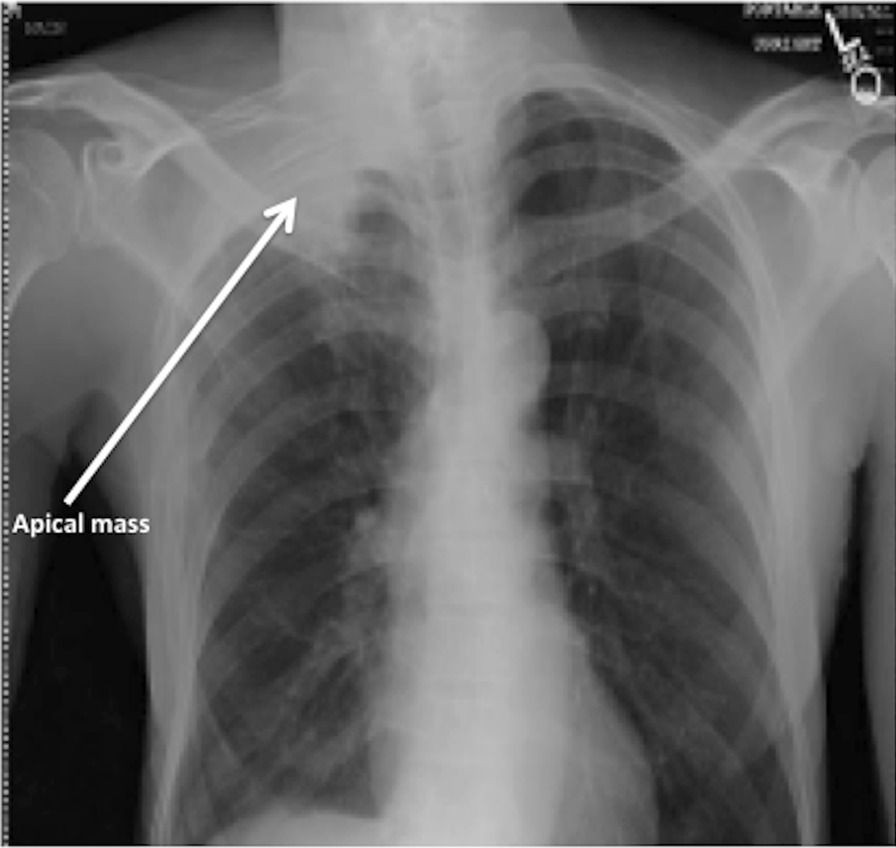
X-ray of the chest: an area of dense opacification in the right apical region; extensive bilateral upper lung bullous changes

Neurological examination revealed joint stiffness, neck pain, arm numbness, arm weakness, and hand weakness. The patient also had restricted shoulder movements and extensive distal and proximal muscle wasting. The right arm was adducted, and the forearm was externally rotated. Muscle strength of the left upper extremity was 5/5; right upper extremity was 2/5, abduction was impaired. Flexion was weak in the wrist, 0/5 on flexion and extension of the fingers. Motor strength of both left lower and right lower extremity was 4+/5. Sensation was decreased in the right upper extremity with complete loss at C8–T1 distribution. Thenar, hypothenar, lumbrical, and interossei muscles were grossly atrophied. The patient also had right-sided miosis. Cerebellar function was normal. His speech was fluent, and cognition was intact. MRI of the cervical spine demonstrated a right apical mass and mild cervical spondylosis (Fig. 1). MRI of the right shoulder showed glenohumeral joint effusion and fullness in the axillary region, consistent with lymphadenopathy (Fig. 1). Chest x-ray showed extensive bullous changes in the right upper lung (Fig. 2). The pulmonary team was consulted, and they recommended chest/pelvic/abdominal CT.

**Fig. 3:**
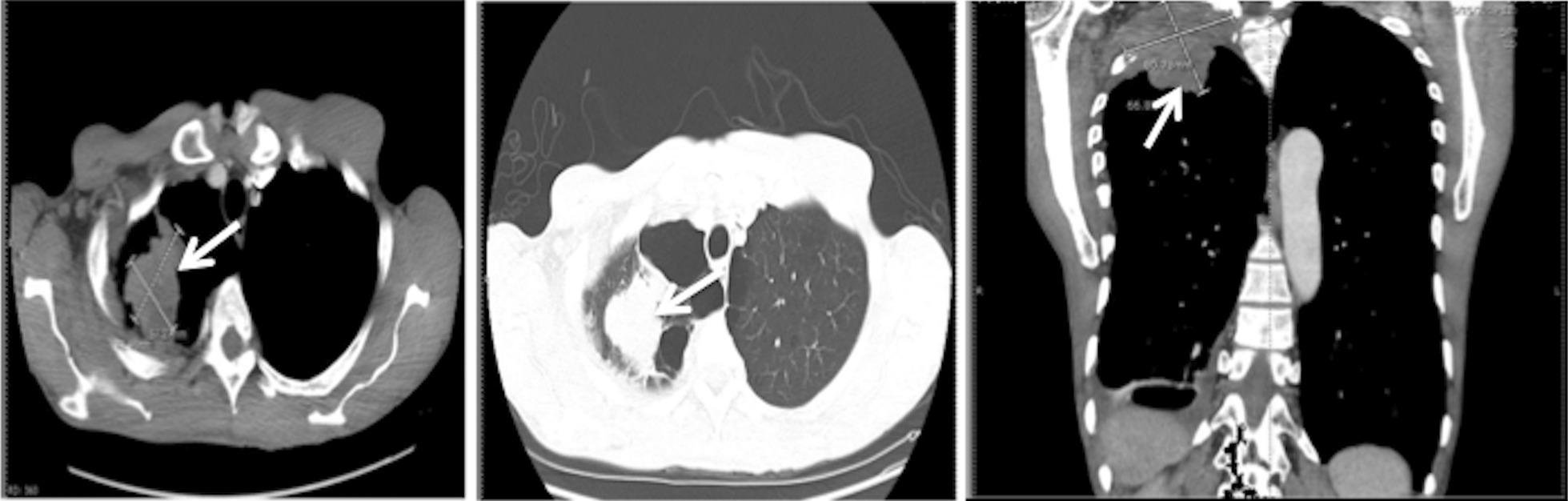
CT images of the chest with and without contrast demonstrating heterogeneous right apical mass causing adjacent rib destruction, extending into the right supraclavicular region (5.2 × 7.6 cm^2^), most likely Pancoast tumor (arrowhead and crosses on CT images)

CT of the chest showed a right apical mass that extended to the right supraclavicular region (Fig. 3). CT-guided biopsy of the apical mass showed non-small cell lung carcinoma. Immunohistochemistry further substantiated the diagnosis of squamous cell carcinoma; the tumor cells expressed CK5, p63, and p40 but were immunonegative for thyroid transcription factor 1 (TTF1), synaptophysin, and chromogranin. Mucicarmine and CK7 immunostaining were diffusely positive. Neuroendocrine differentiation was excluded by the negative synaptophysin and chromogranin.

The oncology and radiation medicine teams were consulted about the patient’s biopsy report. The patient developed superior vena cava syndrome while waiting for the oncology team’s recommendation. He received radiation; the first two fractions were 250 cGy on a 6-mV linear accelerator using a three-dimensional conformal radiotherapy technique. The dose was reduced to 180 cGy for another five fractions afterward. Radiotherapy was completed in 2 weeks. The total dose delivered to the gross tumor volume in the right upper lobe was therefore 1400 cGy. The patient’s pain improved significantly following radiation. The arm swelling and weakness remained the same. Given the tumor size, radiotherapy alone was not effective and subsequent chemotherapy was suggested; risks and benefits of tumor resection were discussed.

## Discussion and conclusions

Pancoast tumors occur in the apical region of the lung, and they usually invade nearby structures including the first and second ribs, brachial plexus, nerves, and blood vessels near the top of the lung, including the subclavian artery, phrenic nerve, recurrent laryngeal nerve, and vagus nerve [4, 7]. One of the factors that leads to delay in diagnosis and treatment of these apical lung cancers is the absence of symptoms from the lung, especially during early clinical presentation. Clinical presentation often varies depending upon the location and type of structures invaded by the tumor at the thoracic inlet. The differential diagnosis may be challenging in the early stages of the disease, and the correct diagnosis is often delayed because the origin of the problem is frequently believed to be orthopedic or rheumatologic in nature [8].

The differential diagnosis for numbness and tingling in patients seen in the neurology clinic can be as little as radiculopathy, from wearing a backpack across the neck and shoulder, to suspicion of a serious diagnosis such as thoracic outlet tumor.

Brachial plexus compression can include tingling sensation, deep pain, and intrinsic hand muscle wasting. Venous thoracic outlet syndrome caused by compression of the neurovascular bundle in the thoracic outlet can cause swelling of the arm and hands, paresthesia, weakness, muscle atrophy, and pain [9, 10].

Lead poisoning and heavy metal radiculopathy were potential differential diagnoses for our patient. His occupation involved working on cars for a collision repair service with longtime exposure to grinding metals for brake pads, car paint fumes, as well as sandblasting. Because his symptoms were asymmetric, the diagnosis of heavy metal radiculopathy was thought to be less likely. Tests for lead and heavy metals were normal.

Our patient had a long history of joint pain and was followed by orthopedics, which may have contributed to his late diagnosis and late referral to the neurology clinic. In addition to weakness of his right hand and grip and the presence of wrist drop, he presented with right pupil miosis, which should alert the physician to a more serious neurological problem. A thorough history and physical examination would have made an earlier diagnosis, and possibly a better clinical outcome, more likely.

It was not initially clear that the patient’s long history of multiple joint pain was related to carcinomatous polyarthritis. This type of paraneoplastic disorder is known to be associated with a variety of tumors, including lung, breast, and gastric [11, 12]. Such disorders can present in a similar fashion to other polyarthritides and may often precede the diagnosis of underlying malignancy [13, 14].

Our patient’s symptoms were consistent with involvement of the brachial plexus, as well as nearby structures. Pancoast tumor can be a challenging diagnosis, but the medical literature shows that earlier diagnosis would have significantly improved the patient’s outcome. The patient had a chest x-ray 5 years prior to the neurology referral, which showed hyperinflated lungs with bullae at the lung apices. A chest x-ray 2 years later showed emphysematous changes, bullous formations, and no acute infiltrates. More recent x-ray showed the appearance of extensive bullous changes in the right upper lung and a mass. Once the diagnosis was established, radiation therapy was initialed and the patient’s pain improved significantly. Given the tumor size, chemotherapy was suggested and risks and benefits of surgical resection were discussed with the patient and his partner. The patient decided against chemotherapy and turned to palliative care.

In summary, aside from shoulder pain, sensory losses in arms, and hand swelling, there were several “red flags” in this patient’s presentation. Chest x-ray, multiple joint pains, and signs of Horner’s syndrome (miosis) should have alerted the treating physician to a more serious problem. Lung cancer is treatable if detected early. An earlier diagnosis could have led to a better outcome for this patient.

## Data Availability

All pertinent data are presented in this case report.
